# SAGRAD: A Program for Neural Network Training with Simulated Annealing and the Conjugate Gradient Method

**DOI:** 10.6028/jres.120.009

**Published:** 2015-06-17

**Authors:** Javier Bernal, Jose Torres-Jimenez

**Affiliations:** 1 National Institute of Standards and Technology, Gaithersburg, MD 20899 USA; 2 CINVESTAV-Tamaulipas, Information Technology Laboratory, Km. 5.5 Carretera Cd., Victoria, Tamaulipas, Mexico

**Keywords:** batch learning, neural networks for classification, scaled conjugate gradient algorithm, simulated annealing

## Abstract

SAGRAD (Simulated Annealing GRADient), a Fortran 77 program for computing neural networks for classification using batch learning, is discussed. Neural network training in SAGRAD is based on a combination of simulated annealing and Møller’s scaled conjugate gradient algorithm, the latter a variation of the traditional conjugate gradient method, better suited for the nonquadratic nature of neural networks. Different aspects of the implementation of the training process in SAGRAD are discussed, such as the efficient computation of gradients and multiplication of vectors by Hessian matrices that are required by Møller’s algorithm; the (re)initialization of weights with simulated annealing required to (re)start Møller’s algorithm the first time and each time thereafter that it shows insufficient progress in reaching a possibly local minimum; and the use of simulated annealing when Møller’s algorithm, after possibly making considerable progress, becomes stuck at a local minimum or flat area of weight space. Outlines of the scaled conjugate gradient algorithm, the simulated annealing procedure and the training process used in SAGRAD are presented together with results from running SAGRAD on two examples of training data.

## 1. Introduction

Neural networks are computational models that work by simulating the way the brain processes information. They are often used to recognize patterns in a data set, say *X*, in Euclidean dimensional *d* − space, *d* some positive integer. Once the neural network is appropriately trained on representative sample patterns of *X*, it can then be used for attempting to recognize other patterns in *X* as they are fed through the network. Accordingly, it is assumed *X* is partitioned into *n* distinct types/classes of patterns, *n* some positive integer.

**Fig. 1:**
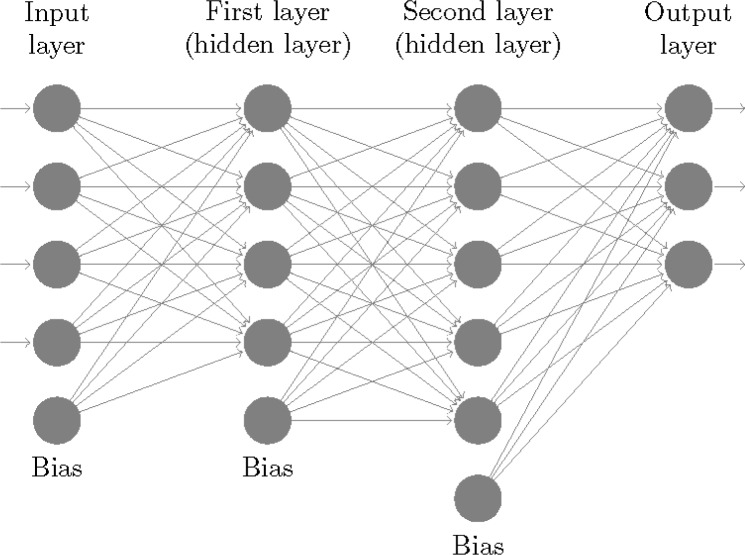
A 4-layer neural network.

Let *A* be a set of training data for *X*, i.e., a subset of *X* in which the *n* distinct types/classes of patterns are well represented. The basic structure of a neural network associated with *X* (to be trained on *A*) consists of layers or columns of mostly computing nodes, or neurons, arranged from left to right (see Fig. 1) in such a way that the result of a computation at each neuron in a layer contributes to the input of neurons in the next layer. The layer at the extreme left of the network is called the input layer of the network (see Fig. 1) and consists of *d* + 1 neurons. A pattern vector in *X*, say *a* = {*a_k_*}, *k* = 1,…,*d*, is introduced into the network through the input layer as follows: *a* is augmented to be of dimension *d* +1 by setting *a_d_*_+1_ equal to 1; neurons in the input layer are labeled with integers from 1 to *d* +1; and for each *k, k* = 1,…,*d* +1, coordinate *a_k_* is assigned to neuron *k* (neuron with label *k*) and as such interpreted to be the output of neuron *k* (neuron *d* +1 is called a bias neuron and its output is 1 for all patterns). The layer immediately to the right of the input layer, unlike the input layer, consists of computing neurons (except for the last neuron which is a bias neuron). From left to right in the network it is the first layer with computing neurons and as such is called the first layer of the network (the layer immediately to the right of this layer is called the second layer of the network, and so on). Like the input layer, the first layer has *d* +1 neurons which are then labeled with integers from *d* + 2 to 2*d* + 2. Given integer *i*, *d* + 2 ≤ *i* ≤ 2*d* +1, a number *x_i_* is designated the input to neuron *i* (in the first layer) which is a weighted sum of the outputs of the input layer (the coordinates of the augmented pattern *a*) expressed as $x_{i}=\sum_{k=1}^{d+1}w_{ki}a_{k}$. Here for each *k*, *k* = 1,…,*d* +1, *w_ki_* is the weight modifying the pattern coordinate *a_k_* before it is fed into neuron *i* (as part of *x_i_*). In order to make neuron *i* into a computing neuron, the sigmoid activation function *σ* (*x*) = 1/(1 + *e*^−^*^x^*) is assigned to it. *σ* is a function with derivatives of all orders and values between 0 and 1. *y_i_* = *σ* (*x_i_*) is then designated the ouput of neuron *i*, *d* + 2 ≤ *i* ≤ 2*d* +1, while *y*_2_*_d_*+_2_ = 1 is designated the output of neuron 2*d* + 2 (the bias neuron). Inductively, given layers *M* and *L*, consecutive layers in the network from left to right; {*y_m_*}, the set of outputs of neurons in layers *M*; *l*_1_, *l*_2_, *l*_1_ < *l*_2_, integers such that neurons in layer *L* are labeled with integers from to *l*_1_ to *l*_2_; and neuron *l*, a neuron in layer *L*, *l*_1_ ≤ *l* ≤ *l*_2_ −1; then a number *x_l_* is designated the input to neuron *l* which is a weighted sum of the outputs of layer *M* expressed as $x_{l}=\sum_{m}w_{ml}y_{m}$. In addition the same sigmoid activation function *σ* defined above is assigned to neuron *l* and *y_l_* =*σ* (*x_l_*) is designated the ouput of neuron *l, l*_1_ ≤ *l* ≤ *l*_2_ −1, while $y_{l_{2}}=1$ is designated the output of neuron *l*_2_ (the bias neuron of layer *L*).

The layer at the extreme right of the network is called the output layer of the network (see Fig. 1). Layers between the input layer and the output layer are called hidden layers (in Fig. 1 the first layer and second layer of the network are the only hidden layers), and hidden layers to the right of the first layer (there is only one, the second layer of the network, in the network of Fig. 1) are all assumed to be of the same length, i.e., to consist of the same number of neurons, a number greater than 1 and preferably greater than *d* and *n*. For consistency with definitions above involving consecutive layers *M* and *L* we assume at first that the output layer contains a bias neuron besides *n* computing neurons. As will become apparent below, there is a one-to-one correspondence between the *n* computing neurons in this layer and the *n* classes of patterns (as defined for *X*) into which the set *A* of training data can be partitioned. After reducing the number of neurons in the output layer to *n* by dropping the dummy bias neuron in the layer, so that for some positive integer *nq*, *nq* is the total number of neurons in the network, neurons in the output layer are then labeled with integers from *nq* − *n* +1 to *nq*. Additionally, letting *nw* be the total number of weights in the network, a natural order can be established for weights so that any given set of *nw* weights can be uniquely identified with a vector, called a weight vector, in weight space, the Euclidean space of dimension *nw*, and vice versa.

Given a pattern *a* in *A*, then for some *q*, 1 ≤ *q* ≤ *n*, *a* is in class *q*, and an *n* – dimensional vector *r*(*a*) = {*r*(*a*)*_m_*}, *m* = 1,…,*n*, called the desired response for *a*, is defined by setting *r*(*a*)*_q_* equal to 1 and *r*(*a*)*_m_* equal to 0 for *m* = 1,…,*n*, *m* ≠ *q*. Another *n* − dimensional vector *o*(*a*) = {*o*(*a*)*_m_*}, *m* = 1,…,*n*, called the actual output for *a*, is defined by setting *o*(*a*)*_m_* equal to the output for *a* of the *m^th^* neuron in the output layer (neuron with label *nq* − *n* + *m*) for each the *m*, *m* = 1,…,*n*. The (total) squared error between desired responses *r*(*a*), and actual outputs *o*(*a*) *a* in *A*, is then

$$E\left(w\right)=1/2\sum_{a\in A}{\left|r\left(a\right)-o\left(a\right)\right|}^{2}=1/2\sum_{a\in A}\sum_{m=1}^{n}{\left(r{\left(a\right)}_{m}-o{\left(a\right)}_{m}\right)}^{2},$$

where *w* is the unique vector in weight space corresponding to the current set of weights in the network. As *E* is implicitly defined in terms of compositions of linear functions between layers in the network and activation functions assigned to neurons in the network, *E* has partial derivatives of all orders at any *w* Accordingly, any optimization method of the gradient kind can be applied for the purpose of hopefully minimizing *E*. If the result of training the neural network on *A*, i.e., minimizing *E* (with gradients, metaheuristics, etc.), is a weight vector *w* at which *E* is zero then it must also be true that the neural network defined by *w* classifies correctly all patterns in the set *A* of training data, i.e., identifies correctly the class to which each pattern belongs. We say then that *w* is a reasonable solution. Additionally, if a subset of *X\A* is also available in which the *n* distinct types/classes of patterns are also well represented, and each pattern in the subset is of known classification, then the neural network defined by *w* should be applied on such a subset for classification results. If the results for a good percentage of the patterns in the subset, say over 90 %, are correct then we say that besides being a reasonable solution, *w* is also a quality solution.

In this paper we discuss SAGRAD, a Fortran 77 program for computing neural networks for classification using batch learning. Classification is one of the most important applications of neural networks. An extensive survey on neural networks for classification can be found in [18]. On the other hand, batch learning is exactly the type of training described above where all patterns in training data are introduced into the network before the training of the network or minimization of the total error *E* begins. This is in contrast with on-line learning where training of the network is done one pattern at a time: each time a pattern in the training data is introduced into the network, training of the network takes place immediately starting at the current solution obtained from introducing the previous pattern, and the training is done only on exactly those patterns, including the current one, that have been introduced into the network so far.

Neural network training in SAGRAD is based on a mixture of simulated annealing [15] and Møller’s scaled conjugate gradient algorithm [7, 9], the latter a variation of the traditional conjugate gradient method [5], better suited for the nonquadratic nature of neural networks. In what follows an outline of Møller’s algorithm is presented that closely resembles the implementation of the algorithm in SAGRAD. In addition, other aspects of the implementation of the training process in SAGRAD are discussed such as the efficient computation of gradients and multiplication of vectors by Hessian matrices that take place in Møller’s algorithm; the (re)initialization of weights with simulated annealing required to (re)start Møller’s algorithm the first time and each time thereafter that it shows insufficient progress in reaching a possibly local minimum; and the use of simulated annealing when Møller’s algorithm, after possibly making considerable progress, becomes stuck at a local minimum or flat area of weight space. Outlines of the simulated annealing procedure and the training process used in SAGRAD are also presented together with results from training with SAGRAD data for two examples. A copy of SAGRAD can be found at http://math.nist.gov/~JBernal.

## 2. Scaled Conjugate Gradient Algorithm

SAGRAD is based on a combination of simulated annealing [15] and Møller’s scaled conjugate gradient algorithm [7, 9] for minimizing the total squared error *E* as a function of weights. Møller’s algorithm, an outline of which is presented below, is based on the well-known conjugate gradient method [5] which works well for quadratic or nearly-quadratic functions. Since the Hessian matrix *E*′′(*w*) of the squared error function *E* at *w* may not be positive definite for *w* in certain areas of weight space, Møller modified the conjugate gradient method based on the approach of the Levenberg-Marquardt algorithm [2]. If at some point during the execution of the conjugate gradient method for some *p* and *w* in *nw* – dimensional Euclidean space *δ* = *p^t^ E*′′(*w*) *p* is computed resulting in a nonpositive *δ*, one makes *δ* positive by adding *p^t^ λ p* to it for some *λ* > 0, i.e., by scaling the Hessian matrix *E*′′(*w*) with the appropriate *λ* > 0 so that *δ* becomes *p^t^* (*E*′′(*w*) +*λI*) *p*, *I* the identity matrix. Once *λ* is initialized it is used and adjusted appropriately throughout the execution of the algorithm so that each *δ* computed as above remains positive. However, since the accuracy of the conjugate gradient method depends on approximating *E*(*w*) with a quadratic function that involves *E*′′(*w*), care must be taken that the scaled *E*′′(*w*) does not produce a bad approximation. This is again taken care of by appropriately raising and lowering *λ*. The outline of the scaled conjugate gradient algorithm below includes the manipulations for raising and lowering *λ*. Here the column vector *E*′(*w*) is the gradient of *E* at weight vector *w*. The outline closely resembles the implementation of Møller’s algorithm in SAGRAD.

1. Initialize weight vector *w*_0_,
  *k* = 0, *ε*_1_ = 10^−6^, *ε*_2_ = 10^−4^, *λ*_0_ = *ε*_2_, ${\bar{\lambda }}_{0}=0$, *r*_0_ = *p*_0_ = −*E*′(*w*_0_), *success* = *true*.
2. Calculate second-order information: *s_k_* = *E*′′(*w_k_*)*p_k_*, $\delta_{k}=p_{k}^{t}s_{k}$.
3. Scale Hessian matrix: $\delta_{k}=\delta_{k}+\left(\lambda_{k}-{\bar{\lambda }}_{k}\right){\left|p_{k}\right|}^{2}$.
4. If *δ_k_* ≤ 0 then scale Hessian matrix to make it positive definite: ${\bar{\lambda }}_{k}=2\left(\lambda_{k}-\delta_{k}/{\left|p_{k}\right|}^{2}\right)$, $\delta_{k}=-\delta_{k}+\lambda_{k}{\left|p_{k}\right|}^{2}$, $\lambda_{k}={\bar{\lambda }}_{k}$.
5. Calculate the step size: $\mu_{k}=p_{k}^{t}r_{k}$, $\alpha_{k}=\mu_{k}/\delta_{k}$.
6. Calculate the comparison parameter Δ*_k_*: ${\bar{w}}_{k+1}=w_{k}+\alpha_{k}p_{k}$, $E\text{\_}q=E\left(w_{k}\right)+E^{\prime }{\left(w_{k}\right)}^{t}\alpha_{k}p_{k}+1/2\left(\alpha_{k}p_{k}^{t}E^{\prime \prime }\left(w_{k}\right)\alpha_{k}p_{k}+\lambda_{k}{\left|\alpha_{k}p_{k}\right|}^{2}\right)$, $\Delta_{k}=\left[E\left(w_{k}\right)-E\left({\bar{w}}_{k+1}\right)\right]/\left[E\left(w_{k}\right)-E\text{\_}q\right]=2\delta_{k}\left[E\left(w_{k}\right)-E\left({\bar{w}}_{k+1}\right)\right]/\mu_{k}^{2}$.
7. Test for error reduction:
  If Δ*_k_* ≥ 0 then a successful error reduction can be made:
$w_{k+1}={\bar{w}}_{k+1}$, $r_{k+1}=-E^{\prime }\left(w_{k+1}\right)$.
  If |*r_k_*_+1_|< *ε*_1_ then terminate and return *w_k_*_+1_ as the desired minimum, perhaps not a global minimum.
  If *success* = *false* or *k* mod *nw* = 0 then restart: *p_k_*_+1_ = *r_k_*_+1_, *λ_k_*_+1_ = *ε*_2_, ${\bar{\lambda }}_{k+1}=0$, *k* = *k* +1, *success* = *true*, and go to step 2.
  Else (if *success* = *true* and *k* mod *nw* ≠ 0) then create new conjugate direction:
$\beta_{k}=\left({\left|r_{k+1}\right|}^{2}-r_{k+1}^{t}r_{k}\right)/\mu_{k}$, $p_{k+1}=r_{k+1}+\beta_{k}p_{k}$.
  If Δ*_k_* ≥ 0.75 then reduce the scale parameter: *λ_k_* = 1/2*λ_k_*.
  Else (if Δ*_k_* < 0) error reduction is not possible:
${\bar{\lambda }}_{k}=\lambda_{k}$, *success* = *false*
  If Δ*_k_* < 0.25 then increase the scale parameter: *λ_k_* = 4*λ_k_*
  if *success* = *false* then go to step 3.
  Else set ${\bar{\lambda }}_{k+1}=0$, *λ_k_*_+1_ = *λ_k_*, *k* = *k* +1, and go to step 2.

## 3. Computing the Gradient

In order to attempt to minimize the error *E* as a function of *w* using the scaled conjugate gradient algorithm as described above, the capability must exist for the efficient computation of the gradient *E*′(*w*) of *E* at *w* and multiplication of a vector by the Hessian matrix *E*′′(*w*) of *E* at *w*. In this section we develop formulas used in SAGRAD for the computation of the gradient *E*′(*w*) as presented in [4]. They originate from the so-called delta rule in [13], [17].

Given *a* ∈ *A*, *w* in weight space, the error at *w* due to *a* is $E_{a}\left(w\right)=1/2{\sum_{m=1}^{n}\left(r{\left(a\right)}_{m}-o{\left(a\right)}_{m}\right)}^{2}$. Thus, $E\left(w\right)=\sum_{a\in A}E_{a}\left(w\right)$. Writing *w* as {*w_k_*}, *k* = 1,…,*nw*, it follows that $\partial E/\partial w_{k}=\sum_{a\in A}\partial E_{a}/\partial w_{k}$ for each *k*, *k* = 1,…,*nw*. Therefore, by fixing *a* in *A*, in what follows it will suffice to develop only the formulas associated with *E_a_*.

As will become apparent from the formulas below, the calculations of the partial derivatives of *E_a_* with these formulas must take place in a specific order, from right to left in the network. This is because each calculation corresponding to a given weight depends on calculations corresponding to other weights in the network to the right of the given weight. Computing in this manner is called backpropagation, originally described in [16]. However, it is an implementation issue that is taken care of in SAGRAD and not necessary for the development of the formulas.

Consider layers *K*, *M*, *L*, consecutive layers in the network from left to right, {*y_k_*}, the set of outputs of neurons in layer *K*, {*y_m_*}, the set of outputs of neurons in layer *M*, and {*x_l_*}, the set of inputs of computing neurons in layer *L*. In particular consider *y_j_*, the output of some neuron in layer *K*, and *x_i_*, *y_i_*, the input and output, respectively, of some computing neuron in layer *M*. In addition, for each *y_k_*, as above let *w_ki_* be the weight such that $x_{i}\sum_{k}w_{ki}y_{k}$ ; and for each *y_m_* and *x_l_* as above let *w_ml_* be the weight such that $x_{l}\sum_{m}w_{ml}y_{m}$.

Case 1. Layer *K* is not the input layer and layer *M* is a hidden layer so that layer *L* is either a hidden layer or the output layer.

Using the chain rule repeatedly we then get

$$\begin{array}{c} \frac{\partial E_{a}}{\partial w_{ji}}=\frac{\partial E_{a}}{\partial x_{i}}\frac{\partial x_{i}}{\partial w_{ji}}=\frac{\partial E_{a}}{\partial x_{i}}\frac{\partial (\sum_{k}w_{ki}y_{k})}{\partial w_{ji}}=\frac{\partial E_{a}}{\partial x_{i}}\frac{\partial (w_{ji}y_{j})}{\partial w_{ji}}=\frac{\partial E_{a}}{\partial x_{i}}y_{j}.\\ \frac{\partial E_{a}}{\partial x_{i}}=\frac{\partial E_{a}}{\partial y_{i}}\frac{\partial y_{i}}{\partial x_{i}}=\frac{\partial E_{a}}{\partial y_{i}}\sigma^{\prime }(x_{i}).\\ \frac{\partial E_{a}}{\partial y_{i}}=\sum_{l}\frac{\partial E_{a}}{\partial x_{l}}\frac{\partial x_{l}}{\partial y_{i}}=\sum_{l}\frac{\partial E_{a}}{\partial x_{l}}\frac{\partial (\sum_{m}w_{ml}y_{m})}{\partial y_{i}}=\sum_{l}\frac{\partial E_{a}}{\partial x_{l}}\frac{\partial (w_{il}y_{i})}{\partial y_{i}}\sum_{l}\frac{\partial E_{a}}{\partial x_{l}}w_{il}. \end{array}$$

Thus,

$$\frac{\partial E_{a}}{\partial w_{ji}}=\frac{\partial E_{a}}{\partial x_{i}}y_{j}=\frac{\partial E_{a}}{\partial y_{i}}\sigma^{\prime }(x_{i})y_{j}=(\sum_{l}\frac{\partial E_{a}}{\partial x_{l}}w_{il})\sigma^{\prime }(x_{i})y_{j}.$$

Case 2. Layer *K* is the input layer so that layer *M* is the first layer in the network. Then {*y_k_*} can be replaced by {*a_k_*}, the set of coordinates of input pattern *a*.

Thus, $x_{i}=\sum_{k}w_{ki}a_{k}$, and

$$\frac{\partial E_{a}}{\partial w_{ji}}=\frac{\partial E_{a}}{\partial x_{i}}a_{j}.$$

Once again

$$\frac{\partial E_{a}}{\partial x_{i}}=\frac{\partial E_{a}}{\partial y_{i}}\sigma^{\prime }(x_{i})$$

and

$$\frac{\partial E_{a}}{\partial y_{i}}=\sum_{l}\frac{\partial E_{a}}{\partial x_{l}}w_{il}.$$

Thus,

$$\frac{\partial E_{a}}{\partial w_{ji}}=\frac{\partial E_{a}}{\partial x_{i}}a_{j}=\frac{\partial E_{a}}{\partial y_{i}}\sigma^{\prime }(x_{i})a_{j}=(\sum_{l}\frac{\partial E_{a}}{\partial x_{l}}w_{il})\sigma^{\prime }(x_{i})a_{j}.$$

Case 3. Layer *M* is the output layer of the network so that there is no layer *L*. Then once again

$$\frac{\partial E_{a}}{\partial w_{ji}}=\frac{\partial E_{a}}{\partial x_{i}}y_{j}$$

and

$$\frac{\partial E_{a}}{\partial x_{i}}=\frac{\partial E_{a}}{\partial y_{i}}\sigma^{\prime }(x_{i}).$$

With {*y_m_*} ordered so that for *m* = 1,…,*n*, *y_m_* = *o*(*a*)*_m_* then $E_{a}(w)=1/2\sum_{m=1}^{n}{\left(r{\left(a\right)}_{m}-o{\left(a\right)}_{m}\right)}^{2}=1/2\sum_{m=1}^{n}{\left(r{\left(a\right)}_{m}-y_{m}\right)}^{2}$, so that

$$\frac{\partial E_{a}}{\partial y_{i}}=y_{i}-r{\left(a\right)}_{i}$$

and

$$\frac{\partial E_{a}}{\partial w_{ji}}=\frac{\partial E_{a}}{\partial x_{i}}y_{j}=\frac{\partial E_{a}}{\partial y_{i}}\sigma^{\prime }(x_{i})y_{j}=(y_{i}-r{\left(a\right)}_{i})\sigma^{\prime }(x_{i})y_{j}.$$

Note that in all cases *σ′*(*x_i_*)= *y_i_* (1 − *y_i_*)

## 4. Fast Exact Multiplication by the Hessian

In this section we develop the formulas used in the implementation of the scaled conjugate gradient algorithm in SAGRAD for the fast exact computation of the product of the Hessian matrix *E*′′(*w*) with an *nw* − dimensional vector *v* in the context of Møller’s algorithm. With these formulas the calculation of the complete Hessian matrix is avoided. These formulas were originally derived by Pearlmutter [12] and Møller [8, 9], and involve the so-called ℛ{·} operator. As in the case of the gradient *E*′(*w*), by fixing *a* in *A*, in what follows it will suffice to develop only the formulas associated with *E_a_*. These formulas depend on the formulas developed above for the computation of the gradient *E*′(*w*), thus simultaneously as *E*′(*w*) is computed with backpropagation, the exact product of *v* and *E*′′(*w*) is computed with these formulas in either a feed-forward fashion or in the manner of backpropagation. But again this is an implementation issue that is taken care of in SAGRAD and not necessary for the development of the formulas.

Let *f* be a differentiable function from *nw* – dimensional Euclidean space into any other finite-dimensional Euclidean space. The ℛ*_v_*{·}operator or simply the ℛ{·}operator is defined by

$${ℛ}_{v}\{f(w)\}\equiv \frac{d}{dr}f(w+rv)|{}_{r=0}=f^{\prime }(w)v,$$

where *f*′(*w*) is the Jacobian matrix of *f* at *w*. In particular ℛ*_v_*{*E*′(*w*)}=*E*″(*w*)*v*. Writing *w* as {*w_k_*}, *k* = 1,…,*nw*, it also follows that for each *k*, *k* = 1,…,*nw*, ℛ*_v_*{∂*E*/∂*w_k_*} is the *k*^th^ component of *E*′′(*w*)*v*.

Given *g*, a differentiable function with domain and range appropriately defined, *c* a real number, then some equations involving ℛ{·} are satisfied:

$$\begin{array}{l} ℛ\{cf(w)\}=cℛ\{f(w)\}\\ ℛ\{f(w)+g(w)\}=ℛ\{f(w)\}+ℛ\{g(w)\}\\ ℛ\{f(w)g(w)\}=ℛ\{f(w)\}g(w)+f(w)ℛ\{g(w)\}\\ ℛ\{g(f(w))\}=g^{\prime }(f(w))ℛ\{f(w)\}\\ ℛ\{\frac{d}{dt}f(w)\}=\frac{d}{dt}ℛ\{f(w)\}\\ ℛ\{w\}=v. \end{array}$$

With these equations and the formulas obtained in the previous section for the components of $E_{a}^{\prime }(w)$, the formulas for the components of $E_{a}^{\prime \prime }(w)v$ can be derived. In what follows weights are doubly indexed. Since there is a one-to-one correspondence between the components of *w* and *v* then the components of *v* will be similarly indexed.

As in the previous section, consider layers *K*, *M*, *L*, consecutive layers in the network from left to right, {*y_k_*}, the set of outputs of neurons in layer *K*, {*y_m_*}, the set of outputs of neurons in layer *M*, and {*x_l_*}, the set of inputs of computing neurons in layer *L.* In particular consider *y_j_*, the output of some neuron in layer *K*, and *x_i_*, *y_i_*, the input and output, respectively, of some computing neuron in layer *M.* In addition, for each *y_k_* as above let *w_ki_* be the weight such that *x_i_* = ∑*_k_w_ki_y_k_*; and for each *y_m_* and *x_l_* as above let *w_ml_* be the weight such that *xl* = ∑*_m_w_ml_y_m_*.

Case 1. Layer *K* is not the input layer and layer *M* is a hidden layer so that layer *L* is either a hidden layer or the output layer.

Applying ℛ{·} on *x_i_* and *y_i_*, we get the feed-forward formulas:

$$\begin{array}{c} ℛ\{x_{i}\}=ℛ\{\sum_{k}w_{ki}y_{k}\}=\sum_{k}ℛ\{w_{ki}y_{k}\}=\sum_{k}\left(ℛ\{w_{ki}\}y_{k}+w_{ki}ℛ\{y_{k}\}\right)=\sum_{k}\left(v_{ki}y_{k}+w_{ki}ℛ\{y_{k}\}\right).\\ ℛ\{y_{i}\}=ℛ\{\sigma (x_{i})\}=\sigma^{\prime }(x_{i})ℛ\{x_{i}\}. \end{array}$$

Applying ℛ{·} on *∂E_a_*/*∂w_ji_*, *∂E_a_*/*∂x_i_*, *∂E_a_*/*∂y_i_*, as computed in the previous section for case 1, we get the backpropagation formulas:

$$\begin{array}{c} ℛ\{\frac{\partial E_{a}}{\partial w_{ji}}\}=ℛ\{\frac{\partial E_{a}}{\partial x_{i}}y_{j}\}=ℛ\{\frac{\partial E_{a}}{\partial x_{i}}\}y_{j}+\frac{\partial E_{a}}{\partial x_{i}}ℛ\{y_{j}\}.\\ ℛ\{\frac{\partial E_{a}}{\partial x_{i}}\}=ℛ\{\frac{\partial E_{a}}{\partial y_{i}}\sigma^{\prime }(x_{i})\}=ℛ\{\frac{\partial E_{a}}{\partial y_{i}}\}\sigma^{\prime }(x_{i})+\frac{\partial E_{a}}{\partial y_{i}}ℛ\{\sigma^{\prime }(x_{i})\}=ℛ\{\frac{\partial E_{a}}{\partial y_{i}}\}\sigma^{\prime }(x_{i})+\frac{\partial E_{a}}{\partial y_{i}}\sigma^{\prime \prime }(x_{i})ℛ\{x_{i}\}.\\ ℛ\{\frac{\partial E_{a}}{\partial y_{i}}\}=ℛ\{\sum_{l}\frac{\partial E_{a}}{\partial x_{l}}w_{il}\}=\sum_{l}ℛ\{\frac{\partial E_{a}}{\partial x_{l}}w_{il}\}=\sum_{l}\left(ℛ\{\frac{\partial E_{a}}{\partial x_{l}}\}w_{il}+\frac{\partial E_{a}}{\partial x_{l}}ℛ\{w_{il}\}\right)=\sum_{l}(ℛ\{\frac{\partial E_{a}}{\partial x_{l}}\}w_{il}+\frac{\partial E_{a}}{\partial x_{l}}v_{il}). \end{array}$$

Case 2. Layer *K* is the input layer so that layer *M* is the first layer in the network. Then {*y_k_*} can be replaced by {*a_k_*}, the set of coordinates of input pattern *a.*

Applying ℛ{·} on *x_i_* and *∂E_a_*/*∂w_i_*, as computed in the previous section for case 2, we get

$$ℛ\{x_{i}\}=ℛ\{\sum_{k}w_{ki}a_{k}\}=\sum_{k}ℛ\{w_{ki}a_{k}\}=\sum_{k}ℛ\{w_{ki}\}a_{k}=\sum_{k}v_{ki}a_{k},$$

and

$$ℛ\{\frac{\partial E_{a}}{\partial w_{ji}}\}=ℛ\{\frac{\partial E_{a}}{\partial x_{i}}a_{j}\}=ℛ\{\frac{\partial E_{a}}{\partial x_{i}}\}a_{j},$$

with the other formulas derived for case 1 above remaining the same.

Case 3. Layer *M* is the output layer of the network so that there is no layer. *L*

Applying ℛ{·} on ∂*E_a_*/∂*y_i_*, as computed in the previous section for case 3, we get

$$ℛ\{\frac{\partial E_{a}}{\partial y_{i}}\}=ℛ\{y_{i}-r{\left(a\right)}_{i}\}=ℛ\{y_{i}\},$$

with the other formulas derived for case 1 above remaining the same.

Note that in all cases *σ″*(*x_i_*) = (1−2*y_i_*)*σ′*(*x_i_*), and *σ′*(*x_i_*) = *y_i_* (1−*y_i_*).

## 5. Simulated Annealing

An outline of the simulated annealing procedure used in SAGRAD follows. It is based on the simulated annealing procedure presented in [15].

In the outline that follows *ε* is a tolerance reasonably chosen (e.g., *ε* = 10^−3^). Accordingly if *w_b_* is the current best solution found by the procedure and the squared error for *w_b_*, i.e., *E_b_=E*(*w_b_*), is less than *ε* then *w_b_* is declared to be a reasonable solution and the procedure is terminated.

1. Input: *w_i_*, *nw*; *w_i_* a weight vector of *nw* coordinates, *nw*>10.
2. Initialize *K*_1_, *K*_2_, *temprture*, *tfactor*, *coef*, *ε* (e.g., *K*_1_=100, *K*_2_=20, *temprture* = 1.0, *tfactor=0.99*, *coef=0.2*, *ε*=10^−3^).
  Set *E_i_* = *E*(*w_i_*), *E_b_* = *E_i_*, *w_c_* = *w_i_*, *E_c_* = ∞, *k*_2_ = 0, *k*_0_ = 0, *k_c_* = 0.
  Initialize *nb* to a positive integer relatively small with respect to *nw* (e.g., *nb* = largest integer ≤ 0.05⋅*nw* if 0.05⋅*nw* ≥ 2, *nb* = 2 otherwise).
3. If *k_c_* = *k*_0_ then set temprture = tfactor ⋅ temprture.
  Set *k*_1_ = 0, *k*_2_ = *k*_2_ +1, *k_c_* = *k*_0_.
4. Set *k*_1_ = *k*_1_ +1, *w_f_* = *w_c_*.
  For *n*=1,…,*nw*, let *w_f_*(*n*) be the *n^th^* coordinate of *w_f_*.
  Generate random integer *nv*, 1 ≤ *nv* ≤ *nb*.
  Generate distinct random integers *j_m_*, *m* = 1,…,*nv*, 1 ≤ *j_m_* ≤ *nw*.
  For each *m*, *m*=1,…,*nv*, generate random number *r*(*m*) in (−1,1), and set
  *w_f_* (*j_m_*) = *w_f_* (*j_m_*) + *coef* ⋅*r*(*m*).
  Set *E_f_* = *E*(*w_f_*).
  If *E_f_*<*E_b_* then set *w_b_=w_f_*, *w_c_* = *w_f_*,*E_b_=E_f_*, *E_c_*=*E_f_*, *k*_0_=*k*_0_+1.
  Else if *Ef* < *E_c_* then set *w_c_* = *w_f_*, *E_c_* = *E_f_*.
  Else if *E_f_* ≥ *E_c_* then generate random number *r* in (0,1), and if $r<{\;\text{exp}}^{\left(E_{c}-E_{f}\right)/temprture}$ then set *w_c_* = *w_f_*, *E_c_* = *E_f_*.
5. If *k*_1_ < *K*_1_ then go to step 4.
6. If *k*_0_ > 0 and *E_b_* < *ε* then go to step 8.
7. If *k*_2_ < *K*_2_ then go to step 3.
8. If *k*_0_>0 then set *w_c_=w_b_*, *E_c_* = *E_b_* (the input solution *w_i_* was improved and *w_c_* now equals the best solution found by the procedure).
9. Output: *w_c_*, *E_c_*, *k*_0_ (if *k*_0_>0 then the input solution *w_i_* was improved and *w_c_* equals the best solution found by the procedure; if *k*_0_=0 then the initial solution *w_i_* was not improved and *w_c_* equals the last solution that was not an improvement but was still accepted by the procedure).

Two versions of the simulated annealing procedure outlined above are used in the training process in SAGRAD. One of low intensity with a relatively small number of iterations, high initial temperature and small neighborhood of exploration (currently with *K*_1_=100, *K*_2_=20, *temprture* = 1.0, *tfactor* = 0.99, *coef* = 0.2, *ε* = 10^−3^), and one of high intensity with a relatively large number of iterations, low initial temperature and large neighborhood of exploration (currently with *K*_1_ = 5000, *K*_2_ = 250, *temprture* = 0.1, *tfactor* = 0.99, *coef* = 1.0, *ε* = 10^−3^). As pointed out in [6], neural network training is typically a two-step process. First, with a method such as the low-intensity version of the simulated annealing procedure mentioned above that tends to elude local minima, weights are initialized. Then an optimization algorithm such as the conjugate gradient method is applied in hopes of finding a global minimum.

In the training process in SAGRAD we follow a slight variation of this two-step strategy while adding an additional step. The additional step involves the high-intensity version of the simulated annealing procedure mentioned above that intensively exploits weight space for a possible global solution. It is used principally when the scaled conjugate gradient algorithm (in the second step), after possibly making considerable progress, becomes stuck at a local minimum or flat area of weight space.

## 6. Training Process

In SAGRAD, neural network training is essentially a three-step process that while still following the two-step strategy in [6], combines in a slightly different manner the two versions of the simulated annealing procedure mentioned above with Møller’s scaled conjugate gradient algorithm. An outline of the training process in SAGRAD in terms of the three steps follows below. There and in what follows a weight vector will be declared to be a reasonable solution if for some reasonably chosen *ε*(e.g., *ε* = 10^−3^), the squared error for the weight vector is less than *ε*. It should also be noted that at different times during the execution of the process, in order to provide the user with the option of getting out of a possibly bad run of the training process, the user will be asked to decide on whether or not to terminate the current run of the training process. If the run is terminated then the user will be asked to decide on whether SAGRAD should stop or do a new cold start of the training process.

In the outline of the training process below note that before the third step is executed (at most once), the first two steps, one following the other, may be executed several times in hopes that the scaled conjugate gradient algorithm in the second step will eventually compute a reasonable solution. The scaled conjugate gradient algorithm in the second step uses as its initial solution the output weights from the last execution of the low-intensity simulated annealing in the first step. On the other hand, the low-intensity simulated annealing in the first step uses as input the best weights found so far among all executions of the scaled conjugate gradient algorithm in the second step except at the start of the execution of the process. At the start of the execution of the process the low-intensity simulated annealing in the first step uses input weights that are randomly generated in the interval (−1,1). It should be noted that all executions of the low-intesity simulated annealing in the first step tend to produce good initial solutions for the scaled conjugate gradient algorithm in the second step. However it is the first execution of the low-intensity simulated annealing in the first step that usually reduces considerably the squared error while all others usually produce no reduction at all. Eventually, as the first two steps are repeatedly executed, either a reasonable solution is found and the process is terminated, or the third step, which involves an execution of the high-intensity simulated annealing followed by an execution of the scaled conjugate gradient algorithm, is executed one time in hopes of finding a reasonable solution. Note that even if a reasonable solution has been found by executing only the first two steps, the user can still direct the training process to go to the third step for a perhaps better solution. Note as well that it may take several cold starts of the training process before a reasonable solution is obtained.

1. (Using low-intensity simulated annealing procedure)
  If at start of execution of process then generate weight vector *w_i_* with coordinates random numbers in the interval (−1,1); set *k*_3_ = 1.
  Otherwise step 2 below and this step have been executed previously:
  set *w_i_* = *w_m_*, where *w_m_* is the best solution found so far among all executions of the scaled conjugate gradient algorithm in step 2 below; set *k*_3_ = *k*_3_ +1.
  With *w_i_* as the input weight vector, execute simulated annealing procedure in previous section using its low-intensity version to produce output weight vector *w_c_*.
2. (Using scaled conjugate gradient algorithm)
  Execute scaled conjugate gradient algorithm with weight vector *w*_0_ in step 1 of the outline of the algorithm in Sec. 2 initialized at *w_c_*.
  At any time during the execution of the algorithm, let *w_m_* be the best solution found so far among all executions in this step, including current one, of the scaled conjugate gradient algorithm. At any time if *w_m_* is a reasonable solution then terminate the training process (unless the user directs the training process to go to step 3 below for a perhaps better solution).
  At any time if “|*r_k_*_+1_ |< *ε*_1_” in step 7 of the outline of the algorithm in Sec. 2 has not occurred (note *r_k_*_+1_ is the negative of the gradient of the squared error function *E* at the current solution *w_k_*_+1_) and *k* > *iter* (e.g, *iter* = 10⋅*nw*, *nw* the number of weights in the network) then if *k*_3_ < *K*_3_ (e.g., *K*_3_ = 20), then go to step 1 above. Otherwise (*k*_3_ ≥ *K*_3_) go to step 3 below. At any time if “|*r_k_*_+1_ |< *ε*_1_” in step 7 of the outline of the algorithm in Sec. 2 has occurred and since *w_m_* is not a reasonable solution so that algorithm is possibly stuck at either a local minimum, i.e., current solution *w_k_*_+1_, or flat area of weight space, then go to step 3 below.
3. (Using high-intensity simulated annealing procedure and scaled conjugate gradient algorithm) Set *w_i_* = *w_m_*, where *w_m_* is the best solution found so far among all executions of the scaled conjugate gradient algorithm in step 2 above.
  With *w_i_* as the input weight vector, execute simulated annealing procedure in previous section using its high-intensity version to produce output weight vector *w_c_*.
  Execute scaled conjugate gradient algorithm with weight vector *w*_0_ in step 1 of the outline of the algorithm in Sec. 2 initialized at *w_c_*.
  At any time during the execution of the algorithm, let *w_m_* be the best solution found so far among this execution and all executions in step 2 above of the scaled conjugate gradient algorithm. At any time if *w_m_* is a reasonable solution, or if “|*r_k_*_+1_|< *ε*_1_” in step 7 of the outline of the algorithm in Sec. 2 has not occurred and *k* > *iter*, or if “|*r_k_*_+1_|< *ε*_1_” in step 7 of the outline of the algorithm in Sec. 2 has occurred, then terminate executions of the scaled conjugate gradient algorithm and the training process. Return *w_m_* as the best solution.

If *w_m_* is not a reasonable solution then the user should direct SAGRAD to do at least one more cold start of the above training process. Even if *w_m_* is a reasonable solution the user can always direct SAGRAD to do more cold starts of the training proces in hopes of getting a better solution.

## 7. Numerical Results

### 7.1 Cushing Syndrome Classification

Here we present results from running SAGRAD on a small example associated with the so-called Cushing syndrome. This is an example used in [3] as an application of neural networks for classification.

The Cushing syndrome is a disorder that occurs when the body is exposed to high levels of the hormone cortisol for a long time. Three types of the syndrome are recognized: adenoma, bilateral hyperplasia, and carcinoma. In the presence of the Cushing syndrome the following observations were made that represent urinary excretion rates (mg/24h) of the steroid metabolites tetrahydrocortisone (in the second column below) and pregnanetriol (in the third column). Each line of observations has a label that appears in the first column, and each of the lines corresponds to an individual identified with each of the observations in the line, an individual with a known type of the syndrome. Accordingly, lines labeled a1, …, a6 correspond to individuals with the adenoma type; lines labeled b1, …, b10 correspond to individuals with the bilateral hyperplasia type; and lines labeled c1, …, c5 correspond to individuals with the carcinoma type. Lines labeled u1, …, u6 correspond to individuals with the syndrome, each individual with an unknown type of the syndrome. Finally, the fourth and fifth columns of the data below have the same data as the second and third columns, respectively, but on a log scale.

**Table:** 

a1	3.1	11.70	1.1314021	2.45958884
a2	3.0	1.30	1.0986123	0.26236426
a3	1.9	0.10	0.6418539	−2.30258509
a4	3.8	0.04	1.3350011	−3.21887582
a5	4.1	1.10	1.4109870	0.09531018
a6	1.9	0.40	0.6418539	−0.91629073
b1	8.3	1.00	2.1162555	0.00000000
b2	3.8	0.20	1.3350011	−1.60943791
b3	3.9	0.60	1.3609766	−0.51082562
b4	7.8	1.20	2.0541237	0.18232156
b5	9.1	0.60	2.2082744	−0.51082562
b6	15.4	3.60	2.7343675	1.28093385
b7	7.7	1.60	2.0412203	0.47000363
b8	6.5	0.40	1.8718022	−0.91629073
b9	5.7	0.40	1.7404662	−0.91629073
b10	13.6	1.60	2.6100698	0.47000363
c1	10.2	6.40	2.3223877	1.85629799
c2	9.2	7.90	2.2192035	2.06686276
c3	9.6	3.10	2.2617631	1.13140211
c4	53.8	2.50	3.9852735	0.91629073
c5	15.8	7.60	2.7600099	2.02814825
u1	5.1	0.40	1.6292405	−0.9162907
u2	12.9	5.00	2.5572273	1.6094379
u3	13.0	0.80	2.5649494	−0.2231436
u4	2.6	0.10	0.9555114	−2.3025851
u5	30.0	0.10	3.4011974	−2.3025851
u6	20.5	0.80	3.0204249	−0.2231436

Log scale data above for observations in lines a1, …, a6, b1, …, b10, c1, …, c5, was used as training data for SAGRAD, and after only one cold start of training process, training was completed on a 4-layer network associated with the data. The input layer of this network had 3 nodes, the first layer 3, the second layer 4, and the ouput layer 3. Then log scale data for observations in lines u1, …, u6, together with the trained network was used to identify with SAGRAD the type (adenoma, bilateral hyperplasia, or carcinoma) corresponding to each of these lines. The classification results from the execution of SAGRAD follow for each line of unknown type. Here the first columm of numbers contains outputs from the ouput node of the neural network corresponding to the ademona type; the second column contains outputs from the output node corresponding to the bilateral hyperplasia type; and finally the third column contains outputs from the output node corresponding to the carcinoma type.

**Table:** 

u1	1.57569381E-25	1.	3.27244446E-61
u2	1.71192768E-10	4.88008365E-06	0.999999999
u3	1.07917885E-38	1.	2.25400938E-41
u4	0.99999641	1.47077866E-05	7.78196988E-08
u5	1.04024021E-38	1.	2.11941796E-41
u6	1.21287938E-38	1.	2.77865859E-41

From these results it appears that adenoma is the type corresponding to line u4; bilateral hyperplasia is the type corresponding to lines u1, u3, u5, u6; and carcinoma is the type corresponding to line u2. This classification of these lines is consistent with the classification of the same lines in [3].

### 7.2 Wine Classification

Data in [1] is the result of a chemical analysis of wines produced in the same region in Italy from three different cultivars. Each line in the data corresponds to a wine and contains quantities of 13 constituents in the wine that were determined through the chemical analysis.

The 13 constituents were:

1. Alcohol
2. Malic acid
3. Ash
4. Alkalinity of ash
5. Magnesium
6. Total phenols
7. Flavanoids
8. Nonflavanoid phenols
9. Proanthocyanins
10. Color intensity
11. Hue
12. OD280/OD315 of diluted wines
13. Proline

Training data for SAGRAD was obtained from [1] as follows. The first 50 lines of data for wine from the first cultivar were extracted from the data and identified as Class 1 training data; the first 60 lines of data for wine from the second cultivar were extracted from the data and identified as Class 2 training data; and the first 40 lines of data for wine from the third cultivar were extracted from the data and identified as Class 3 training data. In all cases each line of data consisted of 13 numbers corresponding in the same order to the quantities of constituents listed above. For example, the first line in the Class 1 training data appeared exactly as follows:

4.23,1.71,2.43,15.6,127,2.8,3.06,.28,2.29,5.64,1.04,3.92,1065

Using this data, SAGRAD was then executed, and after two cold starts of training process, training was completed on a 4-layer network associated with the data. The input layer of this network had 14 nodes, the first layer had 14, the second layer had 15, and the output layer had 3. For the purpose of testing the trained network the remaining 9 lines of data for wine from the first cultivar were extracted from the data and identified as Class 1 independent data; the remaining 11 lines of data for wine from the second cultivar were extracted from the data and identified as Class 2 independent data; and the remaining 8 lines of data for wine from the third cultivar were extracted from the data and identified as Class 3 independent data.

The classification results from the execution of SAGRAD follow for each line of independent data. Here the first columm of numbers contains outputs from the ouput node of the neural network corresponding to wine from the first cultivar; the second column contains outputs from the output node corresponding to wine from the second cultivar; and finally the third column contains outputs from the output node corresponding to wine from the third cultivar. The first 9 lines correspond to the 9 lines in the Class 1 independent data in the same order; the next 11 lines correspond to the 11 lines in the Class 2 independent data in the same order; and the final 8 lines correspond to the 8 lines in the Class 3 independent data in the same order.

0.999999748 1.4638003E-10 3.87972262E-19
0.999999748 1.4638003E-10 3.87972262E-19
0.999999748 1.4638003E-10 3.87972262E-19
0.999999748 1.4638003E-10 3.87972262E-19
0.999999748 1.4638003E-10 3.87972262E-19
0.999999748 1.4638003E-10 3.87972262E-19
0.999999748 1.4638003E-10 3.87972262E-19
0.999999748 1.4638003E-10 3.87972262E-19
0.999999748 1.4638003E-10 3.87972262E-19
0.00725871591 1. 2.44696739E-57
0.999999746 1.47618842E-10 3.86290537E-19
0.00725899153 1. 2.44597986E-57
0.00725948808 1. 2.44420191E-57
0.00725610139 1. 2.45635659E-57
0.0072496458 1. 2.47970923E-57
0.00725948807 1. 2.44420193E-57
0.00725948808 1. 2.44420191E-57
0.00725948808 1. 2.44420191E-57
0.00725948808 1. 2.44420191E-57
3.35969945E-05 1. 1.41314074E-32
2.0606421E-10 5.70930171E-17 1.
2.06064211E-10 5.7093017E-17 1.
2.06064208E-10 5.70930172E-17 1.
2.06064208E-10 5.70930172E-17 1.
2.06065502E-10 5.70929678E-17 1.
2.06064208E-10 5.70930172E-17 1.
2.06064208E-10 5.70930172E-17 1.
2.54410205E-19 1.07524957E-06 1.

From these results it appears that only the wine corresponding to the 2nd line in the Class 2 independent data was classified incorrectly. Additional output from SAGRAD confirms this:

Independent patterns classification results:

Class = 1 Total = 9 Correct = 9 Percentage = 100.
Class = 2 Total = 11 Correct = 10 Percentage = 90.9090909
Class = 3 Total = 8 Correct = 8 Percentage = 100.0

These results compare well with results found elsewhere for the same wine data, e.g., in [10], [11], [14].

## 8. Summary

SAGRAD, a Fortran 77 program for computing neural networks for classification using batch learning, was discussed. Since neural network training in SAGRAD is based in part on Møller’s scaled conjugate gradient algorithm which is a variation of the traditional conjugate gradient method, better suited for the nonquadratic nature of neural networks, an outline of Møller’s algorithm was presented that resembles its implementation in SAGRAD. Important aspects of the implementation of the training process in SAGRAD were discussed such as the efficient computation of gradients and multiplication of vectors by Hessian matrices that are required by Møller’s algorithm. Accordingly, formulas for the product of vectors by Hessian matrices depending on those for the gradients used in SAGRAD were developed. Because of this dependence it was pointed out that calculations with these formulas of the gradient at a vector and the product of the Hessian at the same vector with another vector in the context of Møller’s algorithm occur simultaneously and take place in either a feed-forward fashion or in the manner of backpropagation.

As neural network training in SAGRAD is also based on simulated annealing, an outline of the simulated annealing procedure implemented in SAGRAD was also presented. It was then pointed out that two versions of this procedure are used in the training process in SAGRAD, one a low-intensity version for the (re)initialization of weights required to (re)start the scaled conjugate gradient algorithm the first time and each time thereafter that it shows insufficient progress in reaching a possibly local minimum; and the other a high-intensity version to be used once the scaled conjugate gradient algorithm has possibly reduced the squared error considerably but becomes stuck at a local minimum or flat area of weight space. An outline of the training process was then presented.

Finally, results from executions of SAGRAD were reported. SAGRAD was run on two essentially small examples of training data consisting of sample patterns of dimension 2 and 13, respectively. The trainings with SAGRAD of the training data for the two examples were declared to be good as reasonable solutions were obtained. Classification results were then presented from applying the corresponding neural networks on the trained data, and other independent data of known and unknown classification, and these results were also declared to be good as for over 90 % of the patterns in the data the results were correct.

It should be noted that in general it may take several cold starts of the training process in SAGRAD before a reasonable solution is obtained. Such a solution should then be tested for quality by applying the corresponding neural network on independent sample patterns of known classification. A copy of SAGRAD can be obtained at http://math.nist.gov/~JBernal.
